# Reduced Versus Full-Dose Direct Oral Anticoagulants for Long-Term Management of Venous Thromboembolism: A Systematic Review

**DOI:** 10.3390/jcm15020770

**Published:** 2026-01-17

**Authors:** Manar Al Arifi, Walaa A. Alshahrani, Abdulmajeed M. Alshehri, Majed S. Al Yami

**Affiliations:** 1 Department of Pharmacy Practice, College of Pharmacy, King Saud bin Abdulaziz University for Health Sciences, Riyadh 11481, Saudi Arabia; 2 King Abdullah International Medical Research Center (KAIMRC), Riyadh 11481, Saudi Arabia; alshahraniiwa@gmail.com; 3 Ministry of the National Guard-Health Affairs, Riyadh 14611, Saudi Arabia

**Keywords:** venous thromboembolism (VTE), direct oral anticoagulants (DOACs), reduced-dose anticoagulation, extended-phase therapy, recurrent VTE, major bleeding, clinical outcomes, apixaban, rivaroxaban, long-term management

## Abstract

**Background:** Venous thromboembolism (VTE) is still a serious clinical problem because many patients still have a significant chance of having it happen again after their first course of anticoagulation is over. In recent years, reduced-dose direct oral anticoagulants (DOACs) have been investigated as a means to ensure prolonged protection while diminishing the risk of bleeding complications. This systematic review aims to summarize the available evidence comparing reduced-dose and full-dose DOAC regimens during the extended phase of VTE treatment. **Methods:** A systematic search of PubMed and the Cochrane Library (January 2010–November 2025) identified randomized trials and one ambispective cohort study evaluating reduced-dose apixaban (2.5 mg BID), rivaroxaban (10 mg OD), dabigatran (110 mg BID), or edoxaban (30 mg OD). Methodological quality was assessed using RoB-2 for trials and the Newcastle–Ottawa Scale for observational data. Because of differences in study designs and outcome definitions, a narrative synthesis was applied. **Results:** Five studies met the inclusion criteria. Across trials, reduced-dose DOACs maintained consistently low rates of recurrent VTE: 1.7% in AMPLIFY-EXT versus 8.8% with placebo; 1.2–1.5% in EINSTEIN CHOICE versus 4.4% with aspirin; 2.2% in RENOVE versus 1.8% with full-dose therapy; and 1.3% in HI-PRO versus 10% with placebo. Real-world data from Valeriani et al. showed only a single recurrence (0.7%) over nearly three years. Major bleeding remained uncommon, ranging from 0.1 to 0.5% in randomized trials and 2.1–2.9% in longer-term observational cohorts. **Conclusions:** In summary, reduced-dose DOACs appear to offer a favorable balance of safety and efficacy, providing durable protection against recurrence with a lower bleeding burden. These findings support their role as a practical extended-treatment strategy in clinical practice.

## 1. Introduction

Venous thromboembolism (VTE), including deep vein thrombosis (DVT) and pulmonary embolism (PE), is a major global health problem that contributes significantly to morbidity and mortality and places healthcare systems at risk worldwide. It affects millions of individuals annually and remains one of the leading preventable causes of in-hospital deaths [1]. Although anticoagulants reduce the risk of recurrence during the acute phase of treatment, many patients remain at risk of recurrent VTE after discontinuation of treatment, particularly those with persistent and uncontrolled risk factors [2]. Long-term management decisions must therefore balance the benefits of ongoing anticoagulation against the potential risk of bleeding, requiring individualized assessment of each patient’s clinical profile. This persistent risk has led to the need to develop safe and effective treatment options for long-term treatment. Over the past decade, direct oral anticoagulants (DOACs) have emerged as an alternative to vitamin K antagonists due to their proven pharmacological action, reduced pharmacologic and dietary intervention, and ease of dosing. In recent years, there has been growing interest in evaluating low-dose DOACs as a potential strategy to maintain effective prophylaxis while reducing the risk of bleeding [2]. This approach is especially relevant in patients with moderate thrombotic risk but heightened bleeding concerns, such as the elderly or those with renal impairment. This trend has been explored in several major clinical studies. The AMPLIFY-EXT study evaluated the efficacy of two-dose apixaban for extended treatment [3], the EINSTEIN CHOICE study compared two doses of rivaroxaban and aspirin [4], the RENOVE study compared full-dose and reduced-dose apixaban and rivaroxaban in a multicenter trial [5], the HI-PRO study focused on patients with persistent risk factors [6]. Finally, a recent cohort study provided real-world data supporting the efficacy and safety of long-term treatment with low-dose DOACs [7]. Taken together, the results of these studies indicate a clear evolution in the concept of personalized therapy, where the dose intensity is adjusted based on the balance of clotting risk versus bleeding risk for each patient. This study presents a comparative analysis that summarizes and compares study design, patient characteristics, and baseline clinical outcomes, to provide a comprehensive, evidence-based overview of the extended use of direct oral anticoagulants in the treatment of VTE.

## 2. Materials and Methods

### 2.1. Search Strategy, Study Selection, and Data Extraction

A systematic search was conducted in PubMed and the Cochrane Library to identify studies evaluating the effectiveness and safety of reduced-dose DOACs during the extended-treatment phase of VTE. The reduced-dose regimen for extended therapy is defined as apixaban 2.5 mg twice daily or rivaroxaban 10 mg once daily, as recommended by the CHEST 2021 guidelines, as well as dabigatran 110 mg twice daily or edoxaban 30 mg once daily in selected patients, while the extended treatment phase is described by the ESC 2019 guidelines as anticoagulation continued beyond the initial 3–6 months for long-term secondary prevention [2,8]. The PubMed search strategy combined the terms (“Venous Thromboembolism”[MeSH] OR “Pulmonary Embolism”[MeSH] OR “Deep Vein Thrombosis”[MeSH] OR VTE OR PE OR DVT) AND (“Anticoagulants”[MeSH] OR “Factor Xa Inhibitors”[MeSH] OR “Direct Oral Anticoagulants”[MeSH] OR DOAC* OR apixaban OR rivaroxaban OR dabigatran OR edoxaban) AND (“extended treatment” OR “extended-phase” OR “long-term treatment” OR “reduced-dose” OR “low dose”). A similar search strategy was applied to the Cochrane Library using equivalent keywords. The electronic search covered all records from the introduction of DOACs in 1 January 2010 until 24 November 2025. Only studies published in English were considered.

The eligibility criteria of the included studies were adult patients with objectively confirmed VTE and evaluated reduced-dose DOAC regimens during the extended-treatment phase, reporting at least one clinical outcome of interest, such as recurrent VTE, major bleeding (MB), or clinically relevant non-major bleeding (CRNMB). We included randomized controlled trials and observational cohort studies. Studies were excluded if they were non-original research (systematic reviews, meta-analyses, guidelines, editorials), case reports, conference abstracts, studies not involving reduced-dose DOACs, studies without extractable outcomes, or studies outside the scope of extended-phase anticoagulation.

### 2.2. Quality Assessment and Risk of Bias

**Figure 1:**
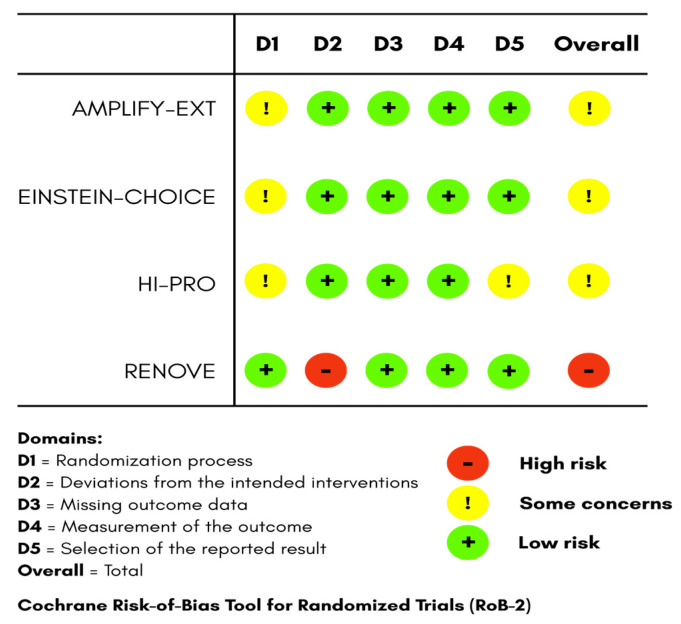
Revised Cochrane Risk-of-Bias Tool for Randomized Trials (RoB-2).

The Revised Cochrane Risk-of-Bias Tool for Randomized Trials (RoB-2) was used to assess risk of bias for randomized controlled trials [9]. It evaluates potential bias across five domains, which includes the randomization process, deviations from intended interventions, missing outcome data, outcome measurement, and selection of the reported result. Each domain was rated as low risk, some concerns, or high risk, and an overall judgment was generated accordingly as shown in Figure 1. On the other hand, Newcastle–Ottawa Scale (NOS) was used for the non-randomized cohort study [10], that assesses the following three main domains: cohort selection (4 points), comparability (2 points), and outcome assessment (3 points). The classification of study quality was based on the total scores (0–9) as low, moderate, or high as shown in Table 1. Using these tools, AMPLIFY-EXT, EINSTEIN CHOICE, and HI-PRO were judged as having some concerns, predominantly due to insufficient reporting of the randomization process [3,4,6]. RENOVE was rated as having high risk of bias owing to its open-label design, which introduced performance-related deviations [5]. Because of strong outcome assessment and sufficient follow-up, Valeriani et al. achieved a moderate-quality NOS rating (6/9 stars); however, it lacked a comparison group [7]. This systematic review is reported according to the Preferred Reporting Items for Systematic Reviews and Meta-Analyses (PRISMA) statement (Table S1) [11]. The study was registered in the International Prospective Register of Systematic Reviews (PROSPERO) with the registration number CRD420251238153.

### 2.3. Data Synthesis and Analysis

Given the heterogeneity in study designs, populations, interventions, and outcome definitions across the included studies, a quantitative meta-analysis was not performed. Instead, a qualitative synthesis was conducted to summarize and compare the evidence. Findings from randomized controlled trials and observational studies were synthesized narratively, with emphasis on study characteristics, methodological quality, outcome definitions, and consistency of reported results. Data from each study were extracted into standardized evidence tables, including population characteristics, intervention and comparator details, outcome measures, effect estimates, and follow-up duration. Studies were grouped and compared according to their design (RCTs vs. cohort study) and the type of anticoagulation regimen evaluated. Where possible, patterns in recurrence risk, bleeding outcomes, and clinical relevance were described. No statistical pooling, subgroup analyses, or sensitivity analyses were undertaken due to the methodological diversity of the included trials.

## 3. Results

### 3.1. Search Results and Study Characteristics

**Figure 2:**
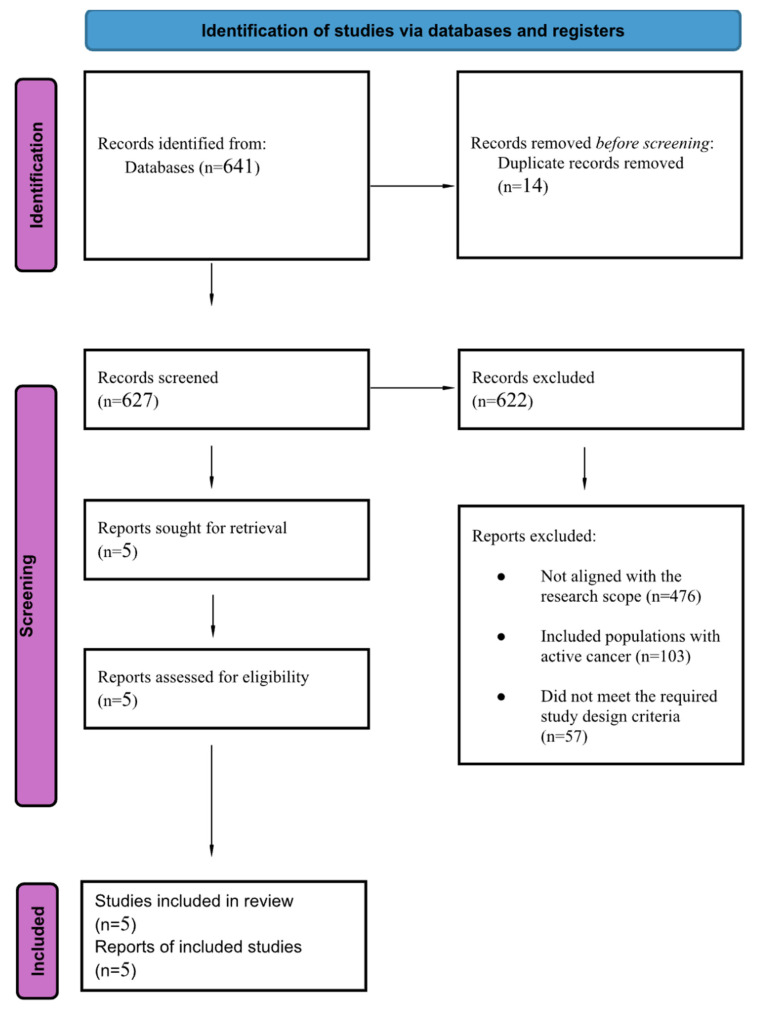
PRISMA flow diagram for selection of studies for the systematic review.

The database search initially yielded 641 records. After automated and manual deduplication, 24 records were identified as potential duplicates, of which 14 were confirmed and removed. A total of 627 unique records remained for screening. Following title and abstract review, the majority of records were excluded for irrelevance to reduced-dose extended anticoagulation in VTE. Full-text assessment was performed for all potentially eligible articles, resulting in the inclusion of five studies [3,4,5,6,7] that met the predefined eligibility criteria for the systematic review as presented in Figure 2. The randomized trials—AMPLIFY-EXT [3], EINSTEIN CHOICE [4], RENOVE [5], and HI-PRO [6]—were mostly multicenter studies that recruited patients from Europe, North America, Australia, and other international sites. Sample sizes varied notably, ranging from 600 participants in HI-PRO to over 3300 in EINSTEIN CHOICE, and the average age across trials generally fell between the mid-50s and early 70s. Women represented roughly one-third to slightly more than half of the enrolled populations. Each trial compared a reduced-dose DOAC regimen—such as apixaban 2.5 mg BID or rivaroxaban 10 mg once daily—with either a full-dose DOAC, aspirin, or placebo. Follow-up periods differed between studies, with AMPLIFY-EXT and HI-PRO following patients for 12 months, while RENOVE extended follow-up to a median of 37 months. Valeriani et al. [7], a single-center ambispective study from Italy, contributed additional real-world data from an older group of patients (mean age 72 years) treated with various reduced-dose DOAC options. Altogether, these studies provide a clear overview of how reduced-dose regimens have been examined across different settings and patient groups. The characteristics of the included studies are presented in Table 2.

### 3.2. Summary of the Included Studies

AMPLIFY-EXT evaluated whether continued apixaban 2.5 mg or 5 mg BID after 6 to 12 months of prior anticoagulation can reduce recurrence without increasing bleeding. It was a randomized, double-blind, placebo-controlled trial comparing apixaban 2.5 mg BID, apixaban 5 mg BID, and placebo for 12 months. Adults ≥ 18 years with confirmed VTE (DVT or PE) who had finished 6 to 12 months of treatment and whose clinicians were uncertain about continuing therapy were enrolled. Efficacy was defined as recurrent symptomatic VTE or VTE-related death, while safety was defined as major bleeding and clinically relevant non-major bleeding. The study excluded patients who had significant renal or hepatic impairment, low hemoglobin or platelet count, or needed other anticoagulants or high-dose antiplatelets, or had any condition that made extended anticoagulation unsafe. Eligible patients were randomized in a 1:1:1 ratio to receive apixaban 2.5 mg BID, apixaban 5 mg BID, or placebo. The use of other anticoagulants and strong CYP3A4/P-gp interacting drugs was prohibited, such as azole antifungals [itraconazole and ketoconazole], macrolide antibiotics [clarithromycin and telithromycin], protease inhibitors [ritonavir, indinavir, nelfinavir, atazanavir, and saquinavir], and nefazadone. The primary efficacy endpoint was recurrent symptomatic VTE or VTE-related death, while the primary safety endpoint was MB. CRNMB was evaluated as a secondary safety outcome. Follow-up occurred regularly during the 12-month treatment period and again 30 days after study completion, with all suspected events confirmed using objective testing. Both apixaban doses (2.5 mg and 5 mg twice daily) produced a major reduction in recurrent VTE or VTE-related death compared with placebo. Recurrent VTE occurred in 14 patients (1.7%) of patients on either apixaban dose, including eight cases of PE (with or without DVT) and six cases of DVT alone versus (8.8%) on placebo. MB did not increase with apixaban (0.1–0.2%) compared with placebo (0.5%). CRNMB was slightly higher with apixaban. Overall, extended therapy with apixaban, especially the 2.5 mg dose, provided strong protection against recurrence with minimal bleeding risk [3].

The EINSTEIN CHOICE trial investigated whether extending anticoagulation with rivaroxaban (20 mg or 10 mg once daily) is superior to aspirin (100 mg once daily) for preventing recurrent VTE in patients who had already completed 6 to 12 months of initial anticoagulant therapy and remained at equipoise regarding continuation of treatment. A total of 3396 patients were randomized, double-blind, phase 3 design. Participants were >18 years old with confirmed proximal DVT or pulmonary embolism. They were eligible only after completing 6 to 12 months of anticoagulation with a VKA or direct oral anticoagulant, without any interruption longer than seven days before randomization. Patients requiring the full dose anticoagulation for other conditions or therapeutic antiplatelet therapy were excluded. Also, exclusions involved severe renal impairment (CrCl < 30 mL/min) or hepatic coagulopathy. Patients were randomized 1:1:1 to receive rivaroxaban 20 mg,10 mg, or aspirin 100 mg once daily for up to year. The primary efficacy outcome was recurrent symptomatic VTE (fatal or nonfatal). Both rivaroxaban doses were more effective than aspirin. Recurrent VTE occurred in 1.5% of patients receiving rivaroxaban 20 mg (0.8% DVT, 0.5% PE and 0.2% fatal venous thromboembolism), 1.2% with rivaroxaban 10 mg (0.6% DVT, 0.4% PE and 0.2% fatal venous thromboembolism), and 4.4% with aspirin. This corresponded to hazard ratios of 0.34 (95% CI, 0.20–0.59) for the 20 mg dose vs. aspirin and 0.26 (95% CI, 0.14–0.47) for the 10 mg dose vs. aspirin. There was no meaningful difference between the two rivaroxaban doses. MB rates were low across all groups: 0.5% for rivaroxaban 20 mg, 0.4% for rivaroxaban 10 mg, and 0.3% for aspirin. CRNMB was slightly higher in the rivaroxaban groups (2.7% for 20 mg and 2.0% for 10 mg) compared with 1.8% in the aspirin group. Subgroup analyses showed that rivaroxaban consistently reduced recurrent VTE in both provoked and unprovoked cases, whereas recurrence was higher with aspirin in both groups. Recurrent VTE rates with aspirin were higher in both provoked (3.6%) and unprovoked (5.6%) cases, while rivaroxaban maintained low recurrence regardless of risk profile. It concluded that both rivaroxaban 20 mg and 10 mg regimens were significantly more effective than aspirin, reducing recurrent VTE by approximately 70%, without an increase in major bleeding. Also, the 10 mg dose offered similar protective effects to the 20 mg treatment dose with less bleeding, making it a preferred option for extended secondary VTE prevention in patients who completed initial anticoagulation [4].

RENOVE trial investigated if a reduced dose of direct oral anticoagulants provides adequate long-term protection against recurrent venous thromboembolism in high-risk patients while lowering bleeding risk compared with full-dose regimens. The study enrolled 2768 adults with confirmed proximal deep-vein thrombosis or pulmonary embolism who had completed 6 months to 2 years uninterrupted of full-dose anticoagulation and were considered to have a high recurrence risk based on persistent risk factors. Patients were randomized to receive either a reduced dose of apixaban 2.5 mg twice daily or rivaroxaban 10 mg once daily or a full dose of apixaban 5 mg twice daily or rivaroxaban 20 mg once daily and were followed for a median of 3.1 years. Recurrent VTE occurred in 2.2% of patients receiving the reduced dose (1.5% PE and 1.1% proximal DVT) and 1.8% of those receiving the full dose (1.5% PE and 1.3% proximal DVT), with an adjusted hazard ratio of 1.32, indicating no clinically meaningful difference in recurrence protection. In contrast, bleeding outcomes showed a clear advantage for the reduced dose strategy. Major or CRNMB occurred far less often in the reduced-dose group, with a five-year cumulative incidence of 9.9%, compared with 15.2% in the full-dose arm. This represented a 39% relative risk reduction and was consistent across all predefined subgroups, regardless of age, sex, renal function, obesity, cancer status, or provoked versus unprovoked index event. Composite outcomes that combined thrombotic and bleeding events also favored the reduced-dose regimen, and overall mortality was slightly lower in the reduced-dose arm, with most deaths attributed to other causes. In conclusion, although noninferiority for the primary endpoint was not achieved due to unexpectedly low recurrence rates, the clinical evidence strongly supports reduced-dose direct oral anticoagulation as an effective and safer long-term strategy for high- risk patients completing extended anticoagulation. The reduced dose provided similar protection against recurrent VTE while significantly lowering bleeding risk and improving overall clinical outcomes. These findings suggest that reduced-dose apixaban or rivaroxaban represents an appropriate preferred option for extended therapy in most high-risk VTE patients [5].

The HI-PRO single-center, randomized, double-blind, placebo-controlled trial evaluated the efficacy and safety of low-intensity extended anticoagulation with apixaban 2.5 mg twice daily for 12 months in adults with provoked VTE who also had at least one enduring risk factor for recurrence. A total of 600 patients were enrolled; all patients had completed at least 3 months of prior anticoagulation for a provoked VTE. Participants were randomized to receive either apixaban 2.5 mg twice daily or placebo for 12 additional months. The primary efficacy outcome was symptomatic recurrent VTE, and the primary safety outcome was the first occurrence of MB according to ISTH criteria. CRNMB and overall adverse events were also assessed. The study demonstrated a substantial reduction in recurrent VTE among patients receiving apixaban. Symptomatic recurrent VTE occurred in only 4 of 300 patients (1.3%) in the apixaban group, compared with 30 of 300 patients (10%) receiving placebo. This corresponded to a hazard ratio of 0.13 with a 95% confidence interval of 0.04 to 0.36 (*p* < 0.001), indicating a marked and statistically significant reduction in risk. No patients receiving apixaban had PE compared to 3.7% in the placebo group. DVT occurred in 1.4% of patients in the apixaban group compared to 7.8% in those who received placebo. These findings highlight the strong protective effect of low-dose apixaban in this specific population, despite their VTE being initially provoked by a clear triggering factor. Safety outcomes were favorable. Major bleeding occurred in just one patient in the apixaban group and in none of the placebo recipients. CRNMB was 4.8% in the apixaban arm versus 1.7% in the placebo arm with no statistically meaningful differences. Subgroup analyses showed consistent benefit across age groups, sex, type of index event (DVT vs. PE), and the nature of provoking and enduring risk factors. In summary, the HI-PRO trial found that one year of extended low-dose apixaban therapy significantly reduced the risk of symptomatic recurrent VTE in patients with provoked VTE who also had at least one ongoing risk factor, without increasing the risk of MB. These results support the use of apixaban 2.5 mg twice daily as an effective and safe extended-therapy strategy for this clinically distinct and previously understudied population [6].

Valeriani et al. is an ambispective single-center cohort study that assessed the long-term effectiveness and safety of reduced-dose DOACs in adults on extended therapy for venous thromboembolism (VTE). A total of 140 patients were enrolled between January 2022–April 2024 after meeting criteria for reduced-dose regimens, which included apixaban 2.5 mg twice daily, rivaroxaban 10 mg daily, dabigatran 110 mg daily, or edoxaban 30 mg daily. All patients had completed at least three months of full-dose anticoagulation, and most index events were lower-extremity DVT, and apixaban was the predominant agent used, followed by rivaroxaban with smaller proportions receiving dabigatran or edoxaban. VTE recurrence during follow-up was exceptionally low at 0.7%, with only one event reported after prolonged apixaban use in a patient with thrombophilia. MB occurred in four patients (2.9%), mainly gastrointestinal or genitourinary, and CRNMB in two patients (1.4%). No fatal bleeding occurred. Across categories, there were no differences in VTE recurrence or bleeding. The study shows that long-term reduced-dose DOAC therapy provides excellent protection against VTE recurrence with low bleeding rates and an acceptable safety profile in an older, comorbid population [7].

### 3.3. Outcomes

Among the included studies, extended anticoagulation consistently reduced the risk of recurrent VTE, with varying safety profiles depending on dose intensity and study design. In AMPLIFY-EXT, recurrent symptomatic VTE or VTE-related death occurred in 1.7% of patients receiving apixaban (both 2.5 mg and 5 mg regimens) (RR 0.97, 95% CI, 0.46–2.02) compared with 8.8% in the placebo group [3]. Similarly, in EINSTEIN CHOICE, rivaroxaban 10 mg and 20 mg resulted in recurrence rates of 1.2% and 1.5%, respectively, (HR 1.34, 95% Cl, 0.65–2.75; *p* = 0.42) vs. 4.4% with aspirin [4]. In the RENOVE trial, recurrent VTE occurred in 2.2% of patients receiving reduced-dose DOACs compared with 1.8% among those receiving full-dose therapy (HR 1.32, 95% CI, 0.67–2.60; *p* = 0.23) [5]. The HI-PRO trial demonstrated a substantial reduction in recurrent VTE with low-dose apixaban (1.3%) compared with placebo 10.0% (HR 0.13, 95% CI, 0.04–0.36; *p* < 0.001) [6]. In the Valeriani et al. study, recurrence was infrequent (0.7%, one patient) during 2.7 years of follow-up in the reduced-dose DOACs [7].

Major bleeding events were uncommon across all studies. In AMPLIFY-EXT, major bleeding occurred in 0.2% and 0.1% of patients treated with apixaban 2.5 mg and 5 mg, respectively (RR 1.93 95% CI, 0.18–21.25), compared with 0.5% in the placebo group [3]. In EINSTEIN CHOICE, major bleeding rates remained low across groups (0.4% with 10 mg rivaroxaban vs. 0.5% with 20 mg rivaroxaban; (HR 1.23, 95% CI, 0.37–4.03; *p* = 0.74) [4]. In RENOVE major bleeding occurred in 2.1% with reduced- dose DOACs and 4% of full-dose DOACs (HR 0.40, 95% CI, 0.22–0.72) [5]. In HI-PRO, major bleeding occurred in only 1 patient receiving low-dose apixaban and in none of the placebo-treated participants [6]. Valeriani et al. reported a major bleeding rate of 2.9% (4 patients) during extended reduced-dose anticoagulation [7].

CRNMB varied slightly by regimen. In AMPLIFY-EXT, CRNMB occurred in 3.0% of the apixaban 2.5 mg group and 4.2% of the 5 mg group, compared with 2.3% for placebo (RR 0.71; 95% CI, 0.43–1.18) [3]. While in EINSTEIN CHOICE, CRNMB occurred 3.3% with 10 mg rivaroxaban vs. 2.4% with 20 mg rivaroxaban (HR 1.37, 95% CI, 0.83–2.26; *p* = 0.21) [4]. However, in RENOVE, CRNMB was significantly lowered in reduced-dose DOACs (8.6%) compared to full-dose DOACs (11.5%) (HR 0.70, 95% CI, 0.53–0.93) [5]. Yet in HI-PRO, CRNMB was observed in 4.8% of low-dose apixaban-treated patients vs. 1.7% of placebo recipients (HR 2.68, 95% CI, 0.96–7.43; *p* = 0.06) [6]. Valeriani et al. reported CRNMB in 1.4% (2 patients) and both were on reduced-dose apixaban [7].

Other clinical outcomes were infrequent. In AMPLIFY-EXT, cardiovascular death, myocardial infarction, or stroke occurred in 0.5% of apixaban 2.5 mg patients vs. 1.3% in placebo [3]. In HI-PRO, non-hemorrhagic and non-fatal adverse events occurred at similarly low rates across treatment groups [6]. Valeriani et al. additionally reported 2.9% arterial thrombotic events (four ischemic strokes) during follow-up [7].

Overall, across randomized and observational evidence, extended low-dose anticoagulation consistently reduced VTE recurrence with low rates of major bleeding and acceptable non-major bleeding profiles, all results summarized in Table 3.

## 4. Discussion

The findings of our systematic review indicate that reduced-dose DOACs provide a favorable balance between long-term efficacy and safety for secondary prevention of venous thromboembolism. In AMPLIFY-EXT, both reduced- and full-dose apixaban maintained similarly low rates of recurrent VTE with minimal bleeding [12]. The EINSTEIN CHOICE trial demonstrated a comparable pattern, showing that rivaroxaban 10 mg was as effective as the full dose while causing no excess major bleeding [4]. Although the RENOVE trial did not formally meet noninferiority criteria, the absolute recurrence rates between reduced- and full-dose arms were nearly identical, with a notable reduction in major and clinically relevant bleeding in the reduced-dose group [5]. Additional support comes from the HI-PRO trial, where low-dose apixaban substantially lowered recurrence compared with placebo and maintained a very low bleeding profile [6]. Real-world findings from the Valeriani et al. study further reinforce the robustness of reduced-dose therapy, showing durable protection over nearly three years of follow-up [7]. Collectively, these results highlight that reduced-dose DOAC strategies can safely maintain long-term protection for most patients requiring extended anticoagulation.

When comparing our findings with the emerging data in cancer-associated VTE, a highly consistent pattern is observed. The EVE trial demonstrated that reduced-dose edoxaban preserved antithrombotic efficacy while significantly lowering major and clinically relevant bleeding among patients with active cancer [12]. Similarly, the API-CAT trial showed that reduced-dose apixaban was noninferior to the full-dose regimen for preventing recurrent VTE in cancer patients, while offering a clinically meaningful reduction in bleeding complications [13]. Despite cancer patients having inherently higher bleeding risks, both trials showed that lowering the anticoagulant intensity did not compromise protection against recurrence. This mirrors the pattern in our review of non-cancer patients, where reduced-dose regimens consistently maintained efficacy with improved safety. Taken together, the alignment between our findings and the EVE and API-CAT trials supports the broader applicability of reduced-dose DOACs across diverse populations, including those with active malignancy.

This review has several strengths. It includes evidence from the major randomized trials investigating extended-phase DOAC therapy as well as real-world data, providing a comprehensive and clinically relevant synthesis. Rigorous methodological tools (RoB-2 and NOS) were applied to assess study quality, and outcomes of direct clinical importance—recurrent VTE, major bleeding, and CRNMB—were systematically compared. The consistency of findings across both controlled trials and observational cohorts strengthens the reliability of our conclusions. However, some limitations should be acknowledged. Heterogeneity across studies—such as differences in population characteristics, DOAC regimens, and follow-up durations—prevented quantitative pooling and necessitated narrative synthesis. The open-label design of the RENOVE trial increases the risk of performance bias, and single-center studies such as HI-PRO and Valeriani et al. may limit generalizability. Additionally, reporting inconsistencies in minor bleeding, arterial events, and subgroup outcomes restricted cross-study comparisons. Finally, restricting our search to English-language publications raises the possibility of language or publication bias. Despite these limitations, the evidence consistently supports the safety and effectiveness of reduced-dose DOACs for extended VTE management.

## 5. Conclusions

In conclusion, our systematic review demonstrates that reduced-dose DOACs represent a safe and effective option for patients requiring extended anticoagulation beyond the initial 3–6 month phase of venous thromboembolism. However, further research is needed to evaluate and confirm these findings using consistent outcome definitions.

## Figures and Tables

**Table 1:** Quality assessment.

Observational Studies Using the Newcastle–Ottawa Scale (NOS)					
Study (Author, Year)	Selection (0–4)	Comparability (0–2)	Outcome (0–3)	Total (0–9)	Quality
Valeriani et al., 2025 [7]	3	0	2	5/9	Good

**Table 2:** Baseline characteristics.

Study	Study Design	Setting/Country Region	Age, Mean/Median (Years)	Sample Size (n)	Female %	Intervention Arm	Follow-Up Duration
AMPLIFY-EXT (2013) [3]	Randomized, double-blind, placebo-controlled	Multicenter, 28 countries (Global)	≈56.7 ± 15.4 years	2486	42–44%	Apixaban 2.5 mg BID vs. Apixaban 5 mg BID vs. Placebo	12 months
EINSTEIN CHOICE (2017) [4]	Randomized, double-blind, active-controlled	Multicenter, 31 countries (Global)	≈57–59 ± 14.7 years	3396	45%	Rivaroxaban 20 mg once daily vs. Rivaroxaban 10 mg once daily vs. Aspirin 100 once daily (control) mg	12 months
RENOVE (2025) [5]	Randomized, open-label, non-inferiority	Multicenter, Europe, Australia, Canada	≈62.7 ± 14.3 years	2768	35%	Reduced-dose DOAC (apixaban 2.5 mg BID or rivaroxaban 10 mg OD) vs. Full-dose DOAC (apixaban 5 mg BID or rivaroxaban 20 mg OD)	Median 37 months (24.0–48.3)
HI-PRO (2025) [6]	Randomized, double-blind, placebo-controlled	Single center, United States (Boston)	59.5 ± 15.2 years	600	57%	Apixaban 2.5 mg BID vs. placebo	12 months
Diagnostics Cohort (2025) [7]	Ambispective (prospective + retrospective) cohort	Single center, Italy	72 ± 15 years	140	52.1%	Reduced-dose DOACs: Apixaban 2.5 mg BID, Rivaroxaban 10 mg OD, Dabigatran 110 mg daily, Edoxaban 30 mg daily	2.7 ± 2.1 years

**Table 3:** Clinical Outcomes.

**AMPLIFY-EXT (2013)** [3]							
**Drug**	**Recurrent VTE (%)**	**Unprovoked (%)**	**Arterial events (%)**	**Major bleeding (%)**	**CRNMB (%)**	**Minor bleeding (%)**	***p*-Value**
Apixaban 2.5 mg BID	1.7%	93.2%	NR	0.2%	3.0%	NR	<0.001
Apixaban 5 mg BID	1.7%	90.7%	NR	0.1%	4.2%	NR	<0.001
Placebo	8.8%	91.1%	NR	0.5%	2.3%	NR	<0.001
**EINSTEIN CHOICE (2017)** [4]							
**Drug**	**Recurrent VTE (%)**	**Unprovoked (%)**	**Arterial events (%)**	**Major bleeding (%)**	**CRNMB (%)**	**Minor bleeding (%)**	***p*-Value**
Rivaroxaban 10 mg OD	1.2%	39.8%	NR	0.4%	2.0%	14.5%	<0.001
Rivaroxaban 20 mg OD	1.5%	42.6%	NR	0.5%	2.7%	11.8%	<0.001
Aspirin 100 mg	4.4%	41.4%	NR	0.3%	1.8%	0.8%	<0.001
**RENOVE (2025)** [5]							
**Drug**	**Recurrent VTE (%)**	**Unprovoked (%)**	**Arterial events (%)**	**Major bleeding (%)**	**CRNMB (%)**	**Minor bleeding (%)**	***p*-Value**
Reduced-dose DOAC	2.2%	60.5%	0.5%	2.1%	8.6%	NR	0.23
Full-dose DOAC	1.8%	61.1%	0.4%	4%	11.5%	NR	0.23
**HI-PRO (2025)** * [6]							
**Drug**	**Recurrent VTE (%)**	**Unprovoked (%)**	**Arterial events (%)**	**Major bleeding (%)**	**CRNMB (%)**	**Minor bleeding (%)**	***p*-Value**
Apixaban 2.5 mg BID	1.3%	0%	0.4%	0.3%	4.8%	NA	<0.001
Placebo	10.0%	0%	0.4%	0%	1.7%	NA	<0.001
**Valeriani et al. (2025)** [7]							
**Drug**	**Recurrent VTE (%)**	**Unprovoked (%)**	**Arterial events (%)**	**Major bleeding (%)**	**CRNMB (%)**	**Minor bleeding (%)**	***p*-Value**
DOAC (single-arm)	0.7%	37.1%	2.9%	2.9%	1.4%	2.1%	—

* HI-PRO exclusively enrolled patients with provoked VTE; unprovoked VTE was an exclusion criterion (Unprovoked = 0%). NR: Not Reported. NA: Not Available.

## Data Availability

No new data were created or analyzed in this study.

## Supplementary Materials

The following supporting information can be downloaded at: https://www.mdpi.com/article/10.3390/jcm15020770/s1, Table S1: PRISMA 2020 Checklist.

## Abbreviations

The following abbreviations are used in this manuscript:

VTE	Venous thromboembolism
DVT	Deep vein thrombosis
PE	Pulmonary embolism
DOAC(s)	Direct oral anticoagulant(s)
VKA(s)	Vitamin K antagonist(s)
MB	Major bleeding
CRNMB	Clinically relevant non-major bleeding
RCT	Randomized controlled trial
ESC	European Society of Cardiology
ISTH	International Society on Thrombosis and Haemostasis
RoB-2	Revised Cochrane Risk-of-Bias tool for randomized trials
NOS	Newcastle–Ottawa Scale
HR	Hazard ratio
RR	Relative Risk
CI	Confidence interval
BID	Twice daily
OD	Once daily
PROSPERO	International Prospective Register of Systematic Reviews
EVE	Extending venous thromboembolism secondary prevention with apixaban in cancer patients trial
API-CAT	Extended Reduced-Dose Apixaban for Cancer-Associated Venous Thromboembolism trial
NR	Not Reported
NA	Not Applicable
